# Somatic Mutation of *PRKAR1A* in Four Cases of Sporadic Cardiac Myxoma

**DOI:** 10.34172/aim.2023.52

**Published:** 2023-06-01

**Authors:** Yunpeng Sun, Zhiping Li, Jingnan Sun, Dashi Ma, Xue Shan, Xia Chen

**Affiliations:** ^1^Department of Cardiac Surgery, The First Hospital of Jilin University, Changchun, Jilin, 130021, China; ^2^Department of Pharmacology, Basic Medical College of Jilin University, Changchun, Jilin, 130021, China; ^3^Department of Hematology, The First Hospital of Jilin University, Changchun, Jilin, 130021, China

**Keywords:** Cardiac myxoma, *PRKAR1A* gene, Somatic mutation, Sporadic

## Abstract

**Background:**

Inactivating mutations of the protein kinase A regulatory subunit 1 alpha (*PRKAR1A*) gene have been reported in familial cardiac myxoma. However, the role of *PRKAR1A* mutation in sporadic cardiac myxoma remains unknown.

**Methods:**

Targeted next-generation sequencing (NGS) was performed to identify mutations with the *PRKAR1A* gene in seven cases of sporadic cardiac myxomas. Sanger sequencing of DNA from cardiac myxoma specimens and matched peripheral blood samples was performed to verify the identified mutations.

**Results:**

Targeted NGS of myxoma DNA revealed 232 single nucleotide variants in 141 genes and 38 insertion-deletion mutations in 13 genes. Six *PRKAR1A* mutations were identified in four of the seven cardiac myxoma cases, and thus, the *PRKAR1A* inactivating mutation rate was 57.2% (4/7, 95% CI=0.44-0.58, *P*<0.05). The *PRKAR1A* variants identified by Sanger sequencing analysis were consistent with those from the NGS analysis for the four myxoma specimens. All of the pathogenic *PRKAR1A* mutations led to premature termination of *PRKAR1A*, except for one synonymous mutation. Moreover, none of the nonsense and missense mutations found in the myxoma specimens were found in the matched peripheral blood samples.

**Conclusion:**

Pathogenic mutations of the *PRKAR1A* gene were identified in tumor specimens from four cases of sporadic cardiac myxoma, and the absence of these mutations in peripheral blood samples demonstrated that they were somatic mutations.

## Introduction

 Cardiac myxoma is the most common primary cardiac neoplasm in adults.^1^ On autopsy, myxomas account for almost half of the cardiac tumors found in 0.17% of the population.^2^ In approximately 75% of the general patient population, this tumor is located in the left atrium and arises from the atrial septum by the stalk.^3,4^ Cardiac myxomas are more likely to occur between the ages of 40 and 60 years.^5^ The clinical symptoms and signs of myxoma include fever of unknown origin, obstruction, and embolization, and although most of them are benign, they can be life-threatening.^6^ To date, the pathogenesis of cardiac myxoma remains unclear.

 The* PRKAR1A* (OMIM 188830) gene encodes the regulatory subunit type 1 alpha (Riα) of the cyclic AMP (cAMP)-dependent protein kinase A (PKA) gene and acts as a tumor suppressor gene.^7,8^ Inactivating mutations ofthe *PRKAR1A* gene are found in familial cases of cardiac myxoma.^9^ Carney complex (CNC) (OMIM 160980) is an inheritable and autosomal dominant condition caused by germline inactivating mutations of the *PRKAR1A *gene.^10,11^ Carney et al first defined CNC as “the syndrome of myxomas, spotty pigmentation, and endocrine overactivity” in 1985.^12^ A pathogenic variant in *PRKAR1A* can be identified and used for diagnosing CNC,^13^ given that inactivating mutations of *PRKAR1A* were shown to be responsible for more than 70% of cases among patients a with familial history of CNC.^14^ More than 100 different mutations throughout the coding region of *PRKAR1A* have been reported.^15^ However, only a relatively small proportion of cardiac myxoma cases are diagnosed as CNC with an established familial pattern of inheritance. Instead, the majority of cardiac myxomas (90%) occur sporadically.^16^ Whether a mutation in the *PRKAR1A* gene is the cause of sporadic cardiac myxomas in patients without a relevant family history and without CNC is still controversial.^4,17,18^ In this study, mutations of *PRKAR1A* were studied in seven cases of sporadic cardiac myxomas that occurred in the absence of a family history of cardiac myxomas and without CNC.

## Materials and Methods

###  Study Participants

 The protocol for this study was reviewed and approved by the Human Medical Ethical Review Committee of the local hospital (The First Hospital of Jilin University, Changchun, China). All the patients were recruited in the Department of Cardiac Surgery, The First Hospital of Jilin University between January 2015 and January 2017 and provided written informed consent for participation. At the onset, the seven patients with cardiac myxomas recruited in this study had no known family history of cardiac myxomas or CNC. Cardiac myxoma specimens and blood samples were collected in cryogenic vials and immediately placed in liquid nitrogen after excision before transfer to a –80°C refrigerator for storage.

###  Histology

 The cardiac myxoma specimens were fixed in 4% paraformaldehyde for 24 hours, washed in phosphate-buffered solution (PBS), and then embedded in paraffin. Hematoxylin and eosin (H&E) staining was used for pathological diagnosis as described previously.^19^ Immunohistochemistry analysis of cardiac myxoma specimens was not performed.

###  Targeted Next-generation Sequencing (NGS) Analysis

 Genomic DNA was isolated using the DNeasy Blood and Tissue Kit (Qiagen, Hilden, Germany). DNA samples were quantified with a Qubit Fluorometer (Thermo Fisher Scientific Inc., Waltham, MA, USA) before the preparation of a library for NGS. The targeted capture genes are listed in Table S1 (Supplementary file 1). Targeted NGS was performed using a Solexa HiSeq 2000 sequencer (Illumina, San Diego, CA, USA). The target genes of the Cancer Risk Cap kit with a hybridization biotin-probe (MyGenostics, Inc, Beijing, China) were hybridized to exon DNA, and formatting was achieved by biotin-streptavidin capture of exon DNA. The sequencing depth was 340 times, and the coverage of the targeted genes was 98.8%. The low-quality reads and 30 and 50 adapters were filtered using Trim Galore (V0.3.5). The filtered reads with a sequencing quality > 20 and read length > 80 were aligned to the reference genome using the Burrows-Wheeler Aligner (V0.7.7).^20^ Duplicate reads were removed using Sequence Alignment/Map tools (V1.1).^15,21^ Single nucleotide variants (SNVs) were identified using UnifiedGenotyper of the GATK (V3.0) program with the dbSNP archive (version 138), using hg19 as the reference genome. Insertion-deletions (INDELs) were identified using the IndelRealigner of GATK (V3.0) and filtered through a 1000-genome database (http://www.1000genomes.org). Genetic variants were annotated across the genome using the ANNOVAR software (V2013Aug23) with respect to mutation type (missense, nonsense, or frameshift) and location (exonic, intronic, or intergenic region).^15,21^ The pathogenicity of the identified variants was assessed according to the “Standards and Guidelines for the Interpretation and Reporting of Sequence Variants in Cancer” published in 2017.^22^

###  Sanger Sequencing Verification

 Sanger sequencing was performed to verify the results of the targeted NGS analysis. Cardiac myxoma samples and matched peripheral blood samples from all seven cases were used for exon capture sequencing. Amplicon sequencing was carried out by Sanger sequencing. The polymerase chain reaction (PCR) primers were designed to produce a product of about 200 bp with the mutation upstream and downstream of each *PRKAR1A* gene. The sequencing primers used are listed in Table S2 (Supplementary file 1).

###  Statistical Analysis 

 The demographic and clinical data are presented as mean ± standard deviation (SD). Patient age, tumor diameter, tumor volume, and genotype frequencies were recorded or calculated, and 95% confidence intervals (CIs) were calculated using SPSS 25.0. Each experiment was repeated at least three times. The Mann-Whitney U test was used for comparisons between two samples. A difference was considered statistically significant with *P* < 0.05.

## Results

###  Clinical Data

**Figure 1:**
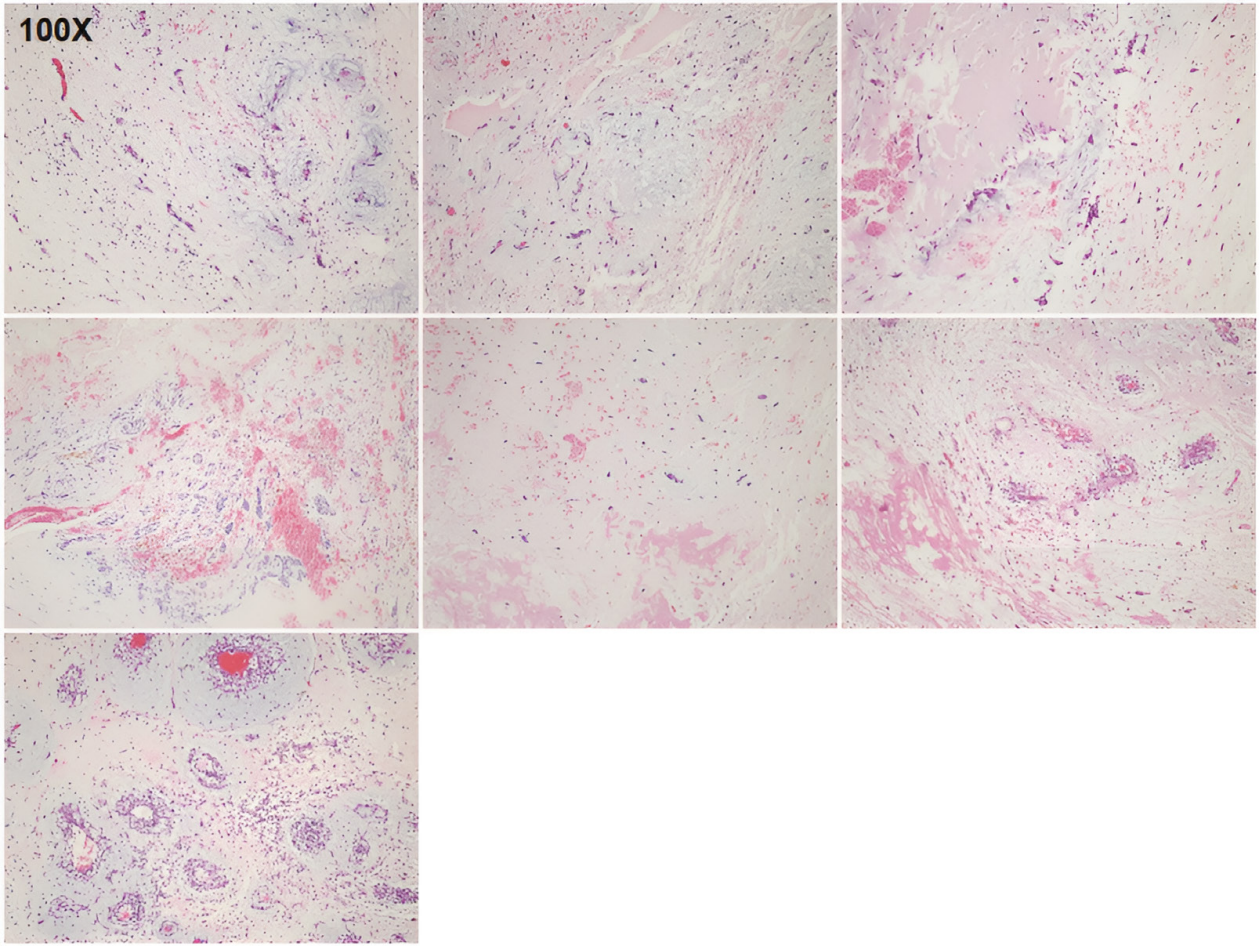
Pathology of Seven Sporadic Cardiac Myxoma Specimens. Hematoxylin and eosin (H&E) staining was performed on sporadic cardiac myxoma samples from seven cases (Case 1 to Case 7). Magnification, 100 ×

 The clinical characteristics of the seven patients with sporadic cardiac myxoma are described in Table 1. Two myxoma patients were male, and five were female. The average age of the patients was 52.85 ± 10.2 years (95% CI = 0.47-0.61 years). The mean age of the women was significantly lower than that of the men (49.0 ± 9.49 years vs 62.5 ± 2.12 years, *P* < 0.05). All seven cardiac tumor specimens exhibited the typical pathological changes of myxoma and were diagnosed as cardiac myxomas (Figure 1). All the myxomas were located on the left side of the atrium. The average tumor diameter was 44 ± 12.47 mm (range, 23–60 mm; 95% CI = 0.32-0.56 mm). Most of the cardiac myxomas were nearly round or oval, and thus, tumor volume was calculated based on a spherical shape. Accordingly, the average tumor volume was 73.26 ± 45.56 cm^3^ (range, 6.37–113.04 cm^3^; 95% CI = 0.41-1.02 cm^3^).

**Table 1:** Clinical Characteristics of Seven Cases with Sporadic Cardiac Myxomas

**No.**	**Gender**	**Age (y)**	**Diameter (mm)**	**Volume (cm**^3^**)**	**Position**
Case 1	Male	64	40	33.49	Atrial septum bottom
Case 2	Female	34	50	65.62	Atrial septum
Case 3	Male	61	60	113.04	Atrial septum
Case 4	Female	56	54	82.41	Atrial septum
Case 5	Female	58	23	6.37	Atrial septum top
Case 6	Female	47	35	22.44	Atrial septum
Case 7	Female	50	46	50.94	Atrial septum bottom

###  Targeted NGS Analysis of Seven Sporadic Cardiac Myxomas

 Using DNA extracted from the seven cardiac myxoma specimens, targeted NGS analysis identified 232 SNVs in 141 genes, which included 87 synonymous mutations, 132 missense mutations, 3 nonsense mutations, and 10 splicing mutations. INDEL variant analysis then demonstrated that 38 gene mutations (3 frameshift mutations and 35 non-frameshift mutations) were distributed in 13 genes (Table S3).

 From the results of this targeted NGS analysis, we highlighted all the genes with a significant number of events. We identified *RAI1* gene mutations in all seven cases, including two synonymous mutations *RAI 1*:c.840G > A (p.Q280Q) and *RAI1*:c.3885G > T(p.P1295P) and five non-frameshift mutations *RAI1*:c.832_834del(p.278_278del), *RAI1*:c.835_837del(p.279_279del), *RAI1*:c.832_837del (p.278_279del),* RAI1*:c.832_843del (p.278_281del), and *RAI1*:c.834_835insCAA (p.Q278delinsQQ). Additionally, eight mutations of the *AR* gene were found in five cases, and all of them led to non-frameshift mutations *AR*:c.170_171insGCA (p.L57delinsLQ), *AR*:c.171_173del (p.57_58del), *AR*:c.173_174insGCAGCA(p.Q58delinsQQQ), *AR*:c.1369_1371del (p.457_457del), *AR*:c.1369_1377del (p.457_459del), *AR*:c.1377_1378insGGC (p.G459delinsGG), *AR*:c.171_179del (p.57_60del), and *AR*:c.170_171insGCAGCA (p.L57delinsLQQ). Therefore, we calculated that the mutation rate of the *AR* gene was 71.4% (5/7; 95% CI = 0.39-0.78). Furthermore, we obtained similar results for non-frameshift mutations from the *TBP* INDEL variants *TBP*:c.163_171del (p.55_57del)and *TBP*:c.223_231 (p.75_77del) in six cases, and the mutation rate was 85.7% (6/7; 95% CI = 0.50-1.00, *P* < 0.05). The *AKAP9* gene mutation involved three missense mutations *AKAP 9*:c.11135G > A (p.R3712Q), *AKAP9*:c.3827G > A (p.R1276Q), and *AKAP9*: c.5725G > A (p.A1909T) and one synonymous mutation *AKAP9*: c.10845G > A (p.K3615K). We identified *AKAP9* gene mutations in four cases, for an *AKAP9* mutation rate of 57.2% (4/7; 95% CI = 0.44-0.58, *P* < 0.05). Five mutations of the *GIGYF2* gene were found among four cases, including one missense mutation *GIGYF 2:*c.3445C > A (p.P1149T), two non-frameshift mutations *GIGYF2*:c.3629_3630insGCA (p.P1210delinsPQ) and *GIGYF2*:c.3611_3612insGCA (p.P1204delinsPQ), and two synonymous mutations* GIGYF2*:c.297T > C (p.A99A)and *GIGYF2*:c.3612A > G (p.P1204P). Therefore, the mutation rate of *GIGYF2 *was also 57.2% (4/7; 95% CI = 0.44-0.58, *P* < 0.05). Still three other genes (*DCTN1*, *GALC* and *GALNS*) were mutated in two cases, and the mutation types were missense mutation, synonymous mutation, and splicing mutation (Table 2).

**Table 2:** Next-generation Sequencing of Multiple Genes in Sporadic Cardiac Myxoma Specimens.

**Gene**	**No.**	**Mutation**	**Amino acid alteration**	**Mutation type**
*RAI1*	Case 1	c.840G > A	p.Q280Q	Synonymous
		c.3885G > T	p.P1295P	Synonymous
		c.832_834del	p.278_278del	Non-frameshift
	Case 2	c.835_837del	p.279_279del	Non-frameshift
	Case 3	c.840G > A	p.Q280Q	Synonymous
		c.832_837del	p.278_279del	Non-frameshift
	Case 4	c.840G > A	p.Q280Q	Synonymous
		c.832_837del	p.278_279del	Non-frameshift
	Case 5	c.832_843	p.278_281del	Non-frameshift
	Case 6	c.840G > A	p.Q280Q	Synonymous
		c.832_834del	p.278_278del	Non-frameshift
	Case 7	c.840G > A	p.Q280Q	Synonymous
		c.832_834del	p.278_278del	Non-frameshift
		c.834_835insCAA	p.Q278delinsQQ	Non-frameshift
*AR*	Case 1	c.170_171insGCA	p.L57delinsLQ	Non-frameshift
	Case 2	c.171_173del	p.57_58del	Non-frameshift
		c.173_174insGCAGCA	p.Q58delinsQQQ	Non-frameshift
		c.1369_1371del	p.457_457del	Non-frameshift
	Case 3	c.171_173del	p.57_58del	Non-frameshift
		c.1369_1377del	p.457_459del	Non-frameshift
		c.1377_1378insGGC	p.G459delinsGG	Non-frameshift
	Case 4	c.171_179del	p.57_60del	Non-frameshift
		c.1369_1371del	p.457_457del	Non-frameshift
	Case 6	c.170_171insGCAGCA	p.L57delinsLQQ	Non-frameshift
		c.1369_1377del	p.457_459del	Non-frameshift
*AKAP9*	Case 1	c.11135G > A	p.R3712Q	Missense
	Case 3	c.10845G > A	p.K3615K	Synonymous
	Case 4	c.3827G > A	p.R1276Q	Missense
	Case 6	c.5725G > A	p.A1909T	Missense
*GIGYF2*	Case 2	c.3629_3630insGCA	p.P1210delinsPQ	Non-frameshift
	Case 3	c.297T > C	p.A99A	Synonymous
		c.3612A > G	p.P1204P	Synonymous
		c.3611_3612insGCA	p.P1204delinsPQ	Non-frameshift
	Case 4	c.3445C > A	p.P1149T	Missense
	Case 6	c.3612A > G	p.P1204P	Synonymous
		c.3611_3612insGCA	p.P1204delinsPQ	Non-frameshift
*TBP*	Case 1	c.163_171del	p.55_57del	Non-frameshift
	Case 2	c.223_231del	p.75_77del	Non-frameshift
	Case 3	c.163_171del	p.55_57del	Non-frameshift
	Case 4	c.163_171del	p.55_57del	Non-frameshift
	Case 6	c.163_171del	p.55_57del	Non-frameshift
	Case 7	c.163_171del	p.55_57del	Non-frameshift
*DCTN1*	Case 1	c.1811A > G	p.Q604R	Missense
	Case 2	c.155C > T	p.P52L	Missense
*GALC*	Case 1	c.645C > T	p.L215L	Synonymous
	Case 3	c.1832T > C	p.L611S	Missense
*GALNS*	Case 2	c.566 + 5T > C	splicing	Splicing
	Case 4	c.566 + 5T > C	splicing	Splicing
*PRKAR1A*	Case 2	c.61_62insAC	p.Y21fs	Frameshift
	Case 3	c.273_274insAAAG	p.V91fs	Frameshift
	Case 6	c.496C > T	p.Q166X	Missense
		c.569delG	p.W190fs	Frameshift
	Case 7	c.366C > G	p.Y122X	Nonsense
		c.678C > T	p.I226I	Synonymous

 Finally, six *PRKAR1A *genemutations were found across four cases, including one missense mutation *PRKAR1 A*:c.496C > T (p.Q166X), one synonymous mutation *PRKAR1A*:c.678C > T (p.I226I), one nonsense mutation *PRKAR1A*:c.366C > G (p.Y122X), and three frameshiftmutations *PRKAR1A*:c.61_62insAC (p.Y21fs), PRKAR1A:c.273_274insAAAG (p.V91fs), and *PRKAR1A*:c.569delG (p.W190fs). The missense mutation, nonsense mutation, and frameshift mutation lead to premature appearance of terminations and eventually give rise to gene mutations (Table 2).

###  PRKAR1A Mutations in Cases of Sporadic Cardiac Myxoma

**Figure 2:**
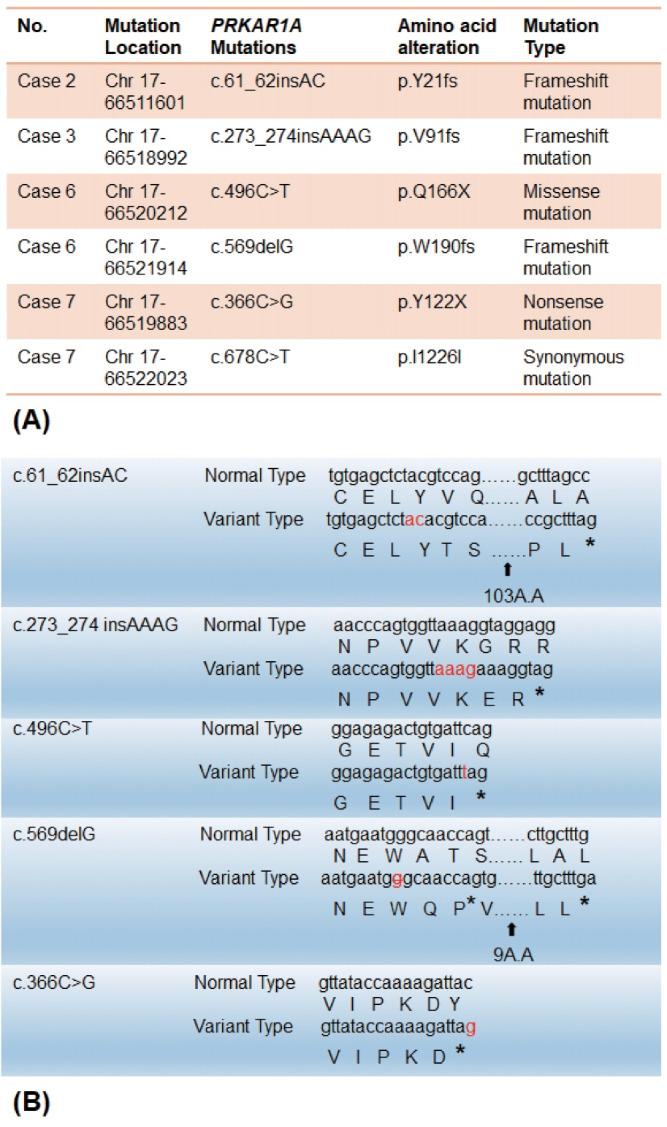
Analysis of the Pathogenic *PRKAR1A *Mutations. **(A)** Targeted next-generation sequencing (NGS) identified six* PRKAR1A* mutations in four cases of sporadic cardiac myxoma. **(B) **Five pathogenic *PRKAR1A* mutations showed a difference between variant type and normal type, whereas one *PRKAR1A* mutation was a synonymous mutation. * indicates the terminators; chr: chromosome

 As described above, targeted NGS detection was performed for the seven cases of sporadic cardiac myxoma. From the analysis of SNVs and INDELs, the following six *PRKAR1A* mutations were detected across four cases: *PRKAR1 A*:c.61_62insAC (p.Y21fs), *PRKAR1A*:c.273_274insAAAG (p.V91fs), *PRKAR1A*:c.496C > T (p.Q166X), *PRKAR1A*:c.569delG (p.W190fs), *PRKAR1A*:c.366C > G (p.Y122X), and *PRKAR1A*:c.678C > T (p.I226I). The three frameshift mutations of* PRKAR1 A*:c.61_62insAC (p.Y21fs), *PRKAR1A*:c.273_274insAAAG (p.V91fs), and *PRKAR1A*:c.569delG (p.W190fs) occurred in the chromosome (Chr) 17 region 66511601 of Case 2, the Chr 17 region 66518992 of Case 3, and the Chr 17 region 66521914 of Case 6 separately. The *PRKAR1 A*:c.678C > T (p.I226I) and *PRKAR1A*:c.366C > G (p.Y122X) mutations were found in the myxoma specimen of Case 7 and were located in the Chr 17 region 66522023 and Chr 17 region 66519883. Notably, the two mutations in Case 7 had differing significance, as one was a synonymous mutation and the other a nonsense mutation. chr17:66520212 *(PRKAR1A )*:c.496C > T (p.Q166X)of Case 6 resulted in a missense mutation (Figure 2a). Because all three types of mutations (nonsense, missense and frameshift) lead to premature termination of *PRKAR1A*, these mutations found in these four cases were *PRKAR1A* pathogenic mutations (Figure 2b). Therefore, the *PRKAR1A* inactivating mutation rate among the seven cases with sporadic cardiac myxomas was 57.2% (4/7; 95% CI = 0.44-0.58). These findings demonstrate that *PRKAR1A* mutation is a pathogenic mechanism of non-familial, sporadic cardiac myxoma in the absence of CNC.

###  Sanger Sequencing Verification in Myxoma Tissue and Matched Peripheral Blood

**Figure 3:**
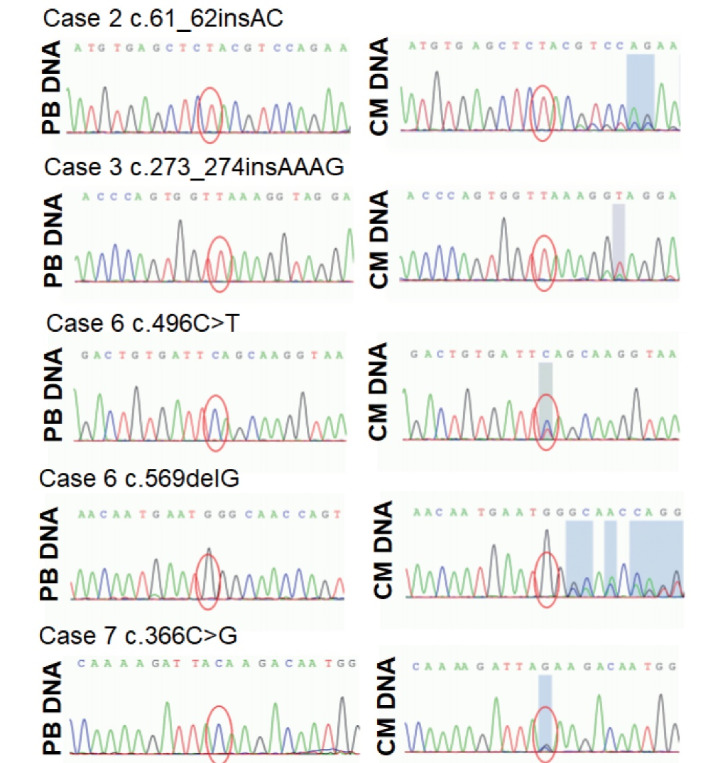
Sanger Sequencing Analysis of Cardiac Myxoma Specimens and Matched Peripheral Blood Samples from the Four Cases with Sporadic Cardiac Myxoma with Pathogenic *PRKAR1A *Mutations Identified by Targeted NGS. Five pathogenic *PRKAR1A* mutations were found in the cardiac myxomas. None of the pathogenic mutations were found in the matched peripheral blood samples. CM, cardiac myxoma; PB, peripheral blood.

 The six mutations of *PRKAR1A* detected in the four cases were verified by Sanger sequencing of both cardiac myxoma specimens and matched peripheral blood samples. Primers were designed to be located upstream and downstream of each *PRKAR1A* mutation (approximately 200 bp in length). Sanger sequencing analysis of myxoma samples identified six *PRKAR1A *mutations that were consistent with the *PRKAR1A* variant mutations detected by targeted NGS analysis (Figure 3). However, no nonsense, missense, or frameshift mutations were found in the matched peripheral blood samples from the four cases, with the exception of one synonymous mutation (c.678C > T) (Figure 3). Because pathogenic mutations in *PRKAR1A* were found in four of the seven (57.2%; 95% CI = 0.44-0.58, *P* < 0.05) cardiac myxoma samples and in none of the matched peripheral blood samples, the *PRKAR1A* mutations in these sporadic cardiac myxomas were considered somatic mutations, which were acquired and non-inheritable.

## Discussion

 The World Health Organization defines cardiac myxoma as a common benign tumor composed of stromal cells scattered in the myxoid matrix.^9^ The incidence of primary cardiac tumors is reported to be 0.0017%–0.19%, approximately 75% of which are benign, and cardiac myxomas account for about 50% of primary benign tumors.^23^ Sporadic cardiac myxomas occur most commonly in middle-aged women, are generally single lesions on the left atrial aspect of the interatrial septum, and do not recur after surgical resection.^24^ In the present study, all seven cardiac myxomas were located on the left atrium, and none of the seven patients had a history of surgery for myxoma or CNC. Although the sample size was small, the number of women was greater than men, and the age of the women was lower than that of the men. The pathological diagnosis of myxoma for all seven sporadic tumors was confirmed by H&E staining (Figure 1).

 DNA was extracted from the seven cardiac myxoma specimens for genetic analysis by targeted NGS. Mutations that were found at a high incidence in these seven cases were compared with the mutations in the Catalogue of Somatic Mutations in Cancer (COSMIC) database (https://cancer.sanger.ac.uk/cosmic). We were surprised to find that the most frequently mutated gene was the *RAI1* gene, with six synonymous mutations and eight non-frameshift mutations occurring in all seven cases. However, in the COSMIC database,* RAI1 *it not a known cancer-related gene. A previous study reported that heterozygous mutations located in the chromosomal region 17p11.2 of *RAI1* cause Smith-Magenis Syndrome (SMS), which is a rare disorder of analgesia and abnormal body clock.^25^ Whether *RAI1 *mutation is related to cardiac myxoma remains to be determined in future studies. Other genes with high mutation rates among the seven cases were *TBP *(6/7), *AR *(5/7), *AKAP9* (4/7), *GIGYF2* (4/7) and *PRKAR1A* (4/7). The *AR* and *TBP *mutations were non-frameshift mutations. The *AR *is a known cancer gene in the COSMIC database, and mutation of *AR* can lead to a variety of androgen-sensitive diseases. *AR* mutations has been shown to be closely related to prostate cancer,^26^ but no association with myxoma has been reported. *AKAP9 *is a putative cancer-related gene in the COSMIC database, but there is currently no evidence that it is associated with myxomatosis. In the COSMIC database, *GIGYF2* is not a known cancer gene, and the missense mutation rate is 51.98%. In the present study, only one missense mutation of *GIGYF2* was identified with no clear relation to myxoma. *DCTN1*, *GALC* and *GALNS *also are not known cancer genes in the COSMIC database. In contrast, *PRKAR1A* is a known cancer gene and also an established gene distributed in myxoma.^9^ The finding in our study that mutation of only *PRKAR1A* could be related to the occurrence of sporadic cardiac myxomas in four cases is of great significance.

 The *PRKAR1A *gene is a tumor suppressor gene that encodes a PKA regulatory 1α subunit.^27^ Previous research has demonstrated that *PRKAR1A* mutation is the cause of CNC in approximately 70% cases and localized on Chr 17 q22-24.^6,28^ However, whether *PRKAR1A* mutation is involved in the pathogenesis of sporadic cardiac myxoma in the absence of a family history or CNC remains a controversial topic. Early studies found no relationship between *PRKAR1A* mutation and sporadic cardiac myxoma morbidity. Fogt et al^29^ analyzed the expression of multiple markers (PRKAR1 9CA, D2S2153, D2S2251 and D2S123) in sporadic cardiac myxoma specimens from 13 patients and found that none showed definite changes in the expression levels of the markers. As a result, they concluded that sporadic cardiac myxomas are not related genetically to CNC. Mantovani et al^30^ investigated the presence of inactivating mutations in *PRKAR1A *in cases of sporadically occurring cardiac myxomas in 29 patients and did not find any mutations by direct sequencing.

 Recent data suggest that the loss of PRKAR1A protein expression may play a role in isolated myxoma tumorigenesis. Maleszewski et al^31^ analyzed the immunohistochemical staining results for 110 cardiac myxoma tissue specimens and observed the absence of *PRKAR1A* antigenicity in all seven cases with CNC and that 32% of isolated cardiac myxomas were similarly nonreactive. *PRKAR1A *gene sequencing analysis in their study further confirmed that 67% of CNC myxomas and 31% of isolated myxomas had pathogenic *PRKAR1A* mutations. Roque et al^18^ presented the case of a 48-year-old Caucasian woman who developed progressive brain metastases one year after removal of a proven sporadic atrial myxoma. Genetic analysis of this case showed multiple mutations within the *PRKAR1A* gene in tissue samples from both the brain metastasis and cardiac tumor, which provides further evidence that *PRKAR1A* mutation can occur in sporadic cardiac myxomas. In the present study, targeted NGS analysis of cardiac myxoma specimens from seven sporadic cases identified five pathogenic mutations of *PRKAR1A *distributed in four sporadic cardiac myxoma cases, for a *PRKAR1A* inactivating mutation rate of 57.2% (4/7). From these results, we conclude that mutation of the *PRKAR1A* gene is one pathogenic mechanism of sporadic cardiac myxoma.

 To further explore the pathogenic role of *PRKAR1A* mutation in sporadic cardiac myxoma, we examined the *PRKAR1A* gene mutations via SNV and INDEL variant analysis. The six *PRKAR1A* mutations found in the four cases included one synonymous mutation (c.678C > T), one nonsense mutation (c.366C > G), one missense mutation (c.496C > T), and three frameshift mutations (c.61_62insAC, c.273_274insAAAG and c.569delG). Yinet al^32^ reported that mice lacking PRKAR1A protein specifically in cardiomyocytes ultimately develop heart failure and myxoma-like phenotype. The majority of *PRKAR1A *defects are premature stop codons generated by nonsense, missense or frameshift (insertions, deletions or splice-site modifications) mutations.^33^ Additionally, the degradation of mutant mRNAs via nonsense-mediated decay was shown to be the cause of *PRKAR1A* haploinsufficiency,^34^ which then activates cAMP signaling and promotes downstream cellular processes that lead to tumorigenesis.^35^ Therefore, the present study demonstrates the occurrence of *PRKAR1A* mutation in myxoma specimens of four sporadic cardiac myxoma cases without CNC or a family history of cardiac myxoma. All of the identified pathogenic mutationsof* PRKAR1A*,which include nonsense, missense and frameshift mutations, lead to premature appearance of terminators (Table 2, Figure 2).

 Inactivating germline mutations of the *PRKAR1A* gene have been reported in the literature in a majority of patients with CNC.^36^ In contrast to the genetic abnormalities observed in CNC patients, few single gene mutations leading to sporadic cardiac myxoma have been found to date.^16,18^ Moreover, the genetic basis of sporadic cardiac myxoma has yet to be fully elucidated. In the present genetic study, we used both cardiac myxoma specimens and matched peripheral blood samples from the same patients for Sanger sequencing analysis. A major discovery of the present study is that all the pathogenic mutations (missense, nonsense, and frameshift mutations) of *PRKAR1A* were found only in the cardiac myxoma samples, with no pathogenic mutations found in the matched peripheral blood samples, except for one synonymous mutation(c.678C > T). Therefore, somatic mutations in the* PRKAR1A* gene may be causative mutations for sporadic cardiac myxoma.

 In conclusion, our study demonstrated that mutation of the *PRKAR1A* gene was associated with the pathogenesis of sporadic cardiac myxoma in four cases lacking a family history of cardiac myxoma or CNC. Notably, the *PRKAR1A* mutations identified in the cardiac myxoma specimens were somatic mutations, which were acquired and non-inheritable.

## Conclusion

 Pathogenic mutations of the PRKAR1A gene were identified in tumor specimens from four cases of sporadic cardiac myxoma, and the absence of these mutations in peripheral blood samples demonstrated that they were somatic mutations.

## Supplementary Files


Supplementary file 1 contains Tables S1-S3.
