# Supplementary material for: Somatic Mutation of PRKAR1A in Four Cases of Sporadic Cardiac Myxoma
Source: Arch Iran Med. 2023 Jun 1;26(6):346–54. doi: 10.34172/aim.2023.52 (PMC10685830; doi:10.34172/aim.2023.52)
Supplement: Supplementary file 1 — contains Tables S1-S3. [file aim-26-346-s001.pdf]

Arch Iran Med. June 2023;26(6):346-S1.

10.34172/aim.2023.52

Supplementaary file 1

Table S1. Targeted Genes of Target-exome Capture Sequencing.

**Targeted genes catalogue of target-exome capture sequencing**					
ABCC6	*CLN3*	*FBN1*	*KCNH2*	*PEX19*	*SLC39A13*
ACE	*COL10A1*	*FBN2*	*KCNJ12*	*PEX7*	*SMARCAL1*
ACP5	*COL11A1*	*FBXO7*	*KCNJ16*	*PIGV*	*SMPD1*
ADAMTSL2	*COL11A2*	*FBXW4*	*KCNQ1*	*PINK1*	*SOST*
AGPS	*COL1A1*	*FERMT3*	*KIF22*	*PITX1*	*SOX9*
AKAP9	*COL2A1*	*FGF10*	*KIF7*	*PRKAG2*	*SPAST*
ALS2	*COL4A2*	*FGFR1*	*KRT17*	*PRKAR1A*	*STAR*
AMH	*COL5A1*	*FGFR2*	*LARS2*	*PRKRA*	*SULF1*
ANK2	*COL9A1*	*FGFR3*	*LIFR*	*PRRT2*	*TAF15*
ANKH	*COL9A2*	*FIG4*	*LMBR1*	*PSEN1*	*TBCE*
ANO5	*COL9A3*	*FLNA*	*LRP5*	*PSEN2*	*TBP*
AR	*COMP*	*FLNB*	*LRRK2*	*PTH1R*	*TBX15*
ARHGAP31	*CSHL1*	*FMN1*	*MAN2B1*	*PTHLH*	*TBX3*
ARSA	*CTSA*	*GAA*	*MAPT*	*PTPN11*	*TBX5*
ARSB	*CTSD*	*GALC*	*MATN3*	*RAI1*	*TBXAS1*
ARSE	*CTSK*	*GALNS*	*MCOLN1*	*RECQL4*	*TCTN3*
ARX	*CUL7*	*GALNT3*	*MGP*	*RIP1*	*THPO*
ASAH1	*CYP11B2*	*GDF5*	*MKS1*	*RMRP*	*TMEM216*
ATP13A2	*CYP21A2*	*GHRHR*	*MMP13*	*RMRPR*	*TMEM67*
ATP6V0A2	*DCTN1*	*GIGYF2*	*MMP9*	*ROR2*	*TNFRSF11A*
ATP7B	*DDR2*	*GLB1*	*MTHFR*	*RPGRIP1L*	*TNNT2*
ATRX	*DHCR24*	*GLI3*	*MYCN*	*RUNX2*	*TP63*
B3GALT6	*DLX3*	*GNPTAB*	*MYH11*	*RYR2*	*TREM2*
BAG3	*DNMT1*	*GPC6*	*MYH7*	*SALL1*	*TRIP11*
BMP2	*DRD2*	*GREM1*	*MYH7*	*SALL4*	*TRPS1*
BMPR1B	*DSC2*	*GSN*	*NAGLU*	*SCN1B*	*TRPV4*
CACNA1S	*DSP*	*HDAC4*	*NEK1*	*SCN3B*	*TYROBP*
CACNB2	*DSPP*	*HEXA*	*NIPBL*	*SCN5A*	*UNC13A*
CCM2	*EIF2AK3*	*HPGD*	*NOTC2*	*SETX*	*WISP3*
CDH3	*ERBB4*	*HSPG2*	*NOTCH2*	*SF1*	*WNT3*
CDKN1C	*ESCO2*	*HTRA1*	*NPR2*	*SGCD*	*WNT5A*
CEP290	*EVC*	*HTRA1*	*OBSL1*	*SH3PXD2B*	*WNT7A*
CHST14	*EVC2*	*ICK*	*PAPSS2*	*SHH*	*WT1*
CHST3	*EXT1*	*IFT122*	*PARK7*	*SHOX*	
CHSY1	*EXT2*	*IFT140*	*PCNT*	*SLC11A2*	

CIZ1	*FAM20C*	*IFT80*	*PEX10*	*SLC25A12*	
CLCN1	*FAM58A*	*IHH*	*PEX14*	*SLC26A2*	
CLCN7	*FBLN1*	*JPH2*	*PEX16*	*SLC35D1*	

Table S2. Primers of Six *PRKAR1A* Mutations Sequencing.

**No.**	***PRKAR1A* Mutations**	**Direction**	**Primer Sequencing**	**Product Length**
Case 2	c.61_62insAC	Forward	TCCCTAGTCCCCACTTCCC	364bp
		Reverse	CACCTCATCATCTCCCCACA	
Case 3	c.273_274insAAAG	Forward	TCCCCTTTGGAATTGGTGTT	465bp
		Reverse	ACCTGTTTTCCAGTACCCAAGA	
Case 6	c.496C>T	Forward	AGGTAGGAACAGGCTCTTTCT	448bp
		Reverse	ACAAAGTGTTCTGTCCATCTCAGA	
	c.569delG	Forward	TGCTCAGCACGGTTCTCTA	447bp
		Reverse	TAGCTGTCTCGGTCGATGC	
Case 7	c.366C>G	Forward	GGCTGTAGGCAAGGGGATT	489bp
		Reverse	AAGAGAGAAGGCCTCGTGT	
	c.678C>T	Forward	TCTCTGTTGTGTACTGCAAACA	476bp
		Reverse	AGCTGGGCTTAATGCAAAGT	

Table S3. Variants Detected by Targeted NGS in Seven Cases of CM.

No.	Variants Type	Genes	Mutations	Amino acid change	Variant type
Case 1	SNVs	*AKAP9*	c.11135G>A	p.R3712Q	Missense mutation
	SNVs	*AMH*	c.1165G>T	p.E389X	Nonsense mutation
	SNVs	*ANK2*	c.7117A>G	p.T2373A	Missense mutation
	SNVs	*ARX*	c.612C>T	p.R204R	Missense mutation
	SNVs	*ASAH1*	c.91A>G	p.I31V	Missense mutation
	SNVs	*CACNA1S*	c.1143C>T	p.F381F	Missense mutation
	SNVs	*CEP290*	c.6787A>G	p.S2263G	Missense mutation
	SNVs	*CLCN7*	c.2343G>A	p.T781T	Synonymous mutation
	SNVs	*COL4A2*	c.4255A>G	p.M1419V	Missense mutation
	SNVs		c.4256T>C	p.M1419T	Missense mutation
	SNVs		c.1035G>C	p.G345G	Synonymous mutation
	SNVs	*CTSA*	c.1047G>C	p.K349N	Missense mutation
	SNVs	*DCTN1*	c.1811A>G	p.Q604R	Missense mutation
	SNVs	*DLX3*	c.736G>C	p.D246H	Missense mutation
	SNVs	*DSP*	c.1481A>T	p.Y494F	Missense mutation
	SNVs	*DYNC2H1*	c.7143A>G[1]	p.V2381V	Synonymous mutation
	SNVs	*ERBB4*	c.1284C>T	p.L428L	Synonymous mutation
	SNVs	*EVC2*	c.2155G>C	p.D719H	Missense mutation
	SNVs		c.2154G>A	p.R718R	Synonymous mutation
	SNVs	*EVC*	c.1207G>A	p.G403S	Missense mutation
	SNVs	*FBLN1*	c.1978G>A	p.V660M	Missense mutation
	SNVs	*FBXO7*	c.1268A>G	p.N423S	Missense mutation
	SNVs	*FERMT3*	c.1914G>C	p.S638S	Synonymous mutation
	SNVs	*FGFR3*	c.990C>T	p.T330T	Synonymous mutation
	SNVs	*FLNB*	c.1249G>A	p.V417M	Missense mutation
	SNVs	*FMN1*	c.99A>G	p.S33S	Synonymous mutation
	SNVs	*GALC*	c.645C>T	p.L215L	Synonymous mutation
	SNVs	*GHRHR*	c.47C>T	p.P16L	Missense mutation
	SNVs	*GNPTAB*	c.2504C>T	p.P835L	Missense mutation
	SNVs	*HEXB*	c.1279A>G	p.S427G	Missense mutation
	SNVs	*HPGD*	c.476A>G	p.N159S	Missense mutation
	SNVs	*HSPG2*	c.12742G>T	p.G4248C	Missense mutation
	SNVs	*HTRA1*	c.289G>A	p.A97T	Missense mutation
	SNVs	*KCNQ1*	c.1343C>G	p.P448R	Missense mutation

	SNVs	*LRP5*	c.63G>A	p.A21A	Synonymous mutation
	SNVs	*LRRK2*	c.7153G>A	p.G2385R	Missense mutation
	SNVs	*MMP9*	c.113A>G	p.N38S	Missense mutation
	SNVs	*MTHFR*	c.136C>T	p.R46W	Missense mutation
	SNVs	*OBSL1*	c.569C>G	p.A190G	Missense mutation
	SNVs	*PINK1*	c.857C>T	p.P286L	Missense mutation
	SNVs	*RAI1*	c.840G>A[1]	p.Q280Q	Synonymous mutation
	SNVs		c.3885G>T	p.P1295P	Synonymous mutation
	SNVs	*SDHA*	c.163T>C	p.Y55H	Missense mutation
	SNVs	*SHH*	c.555G>A	p.V185V	Synonymous mutation
	SNVs	*TNNT2*	c.68-5C>T	splicing	Splicing mutation
	SNVs	*TRPV4*	c.139C>G	p.L47V	Missense mutation
	SNVs	*TYROBP*	c.68G>A	p.R23H	Missense mutation
	SNVs	*VPS54*	c.409T>C	p.L137L	Synonymous mutation
	INDELs	*AR*	c.170_171insGCA	p.L57delinsLQ	non-frameshift mutation
	INDELs	*CDKN1C*	c.512_523del	p.171_175del	non-frameshift mutation
	INDELs	*RAI1*	c.832_834del	p.278_278del	non-frameshift mutation
	INDELs	*TBP*	c.163_171del	p.55_57del	non-frameshift mutation
Case 2	SNVs	*CCM2*	c.631G>A	p.V211M	Missense mutation
	SNVs	*CIZ1*	c.424C>T	p.R142C	Missense mutation
	SNVs	*COL11A2*	c.1039C>G	p.R347G	Missense mutation
	SNVs	*DCTN1*	c.155C>T	p.P52L	Missense mutation
	SNVs	*FBN2*	c.3518C>G	p.T1173S	Missense mutation
	SNVs	*GALNS*	c.566+5T>C	splicing	Splicing mutation
	SNVs	*HDAC4*	c.563A>G	p.H188R	Missense mutation
	SNVs	*ICK*	c.527A>G	p.N176S	Missense mutation
	SNVs	*KRT17*	c.1045G>A	p.V349M	Missense mutation
	SNVs	*LRP5*	c.3361A>G	p.N1121D	Missense mutation
	SNVs	*MTHFR*	c.665C>T[1]	p.A222V	Missense mutation
	SNVs	*NOTCH2*	c.137A>G	p.N46S	Missense mutation
	SNVs		c.112G>A	p.E38K	Missense mutation
	SNVs	*PCNT*	c.3735C>A	p.S1245R	Missense mutation
	SNVs	*PRRT2*	c.439G>C	p.D147H	Missense mutation
	SNVs	*STAR*	c.16T>C	p.F6L	Missense mutation
	SNVs	*TRIP11*	c.1250A>G	p.N417S	Missense mutation
	INDELs	*AR*	c.171_173del	p.57_58del	non-frameshift mutation
	INDELs		c.173_174insGCAGCA	p.Q58delinsQQQ	non-frameshift mutation

	INDELs		c.1369_1371del	p.457_457del	non-frameshift mutation
	INDELs	*GIGYF2*	c.3629_3630insGCA	p.P1210delinsPQ	non-frameshift mutation
	INDELs	*LRP5*	c.33_38del	p.11_13del	non-frameshift mutation
	INDELs	*PRKAR1A*	c.61_62insAC	p.Y21fs	Frameshift mutation
	INDELs	*RAI1*	c.835_837del	p.279_279del	non-frameshift mutation
	INDELs	*SALL1*	c.477_478insAGC	p.G160delinsSG	non-frameshift mutation
	INDELs	*TBP*	c.223_231del	p.75_77del	non-frameshift mutation
Case 3	SNVs	*ABCC6*	c.955A>G	p.I319V	Missense mutation
	SNVs	*AKAP9*	c.10845G>A	p.K3615K	Synonymous mutation
	SNVs	*CIZ1*	c.1049C>T	p.A350V	Missense mutation
	SNVs	*CTSD*	c.957G>A	p.P319P	Synonymous mutation
	SNVs	*DLX3*	c.736G>C	p.D246H	Missense mutation
	SNVs	*DNMT1*	c.410C>G	p.T137R	Missense mutation
	SNVs	*DSP*	c.3646A>G	p.I1216V	Missense mutation
	SNVs	*DYNC2H1*	c.12218T>C	p.L4073P	Missense mutation
	SNVs	*ERBB4*	c.1177C>T	p.R393W	Missense mutation
	SNVs	*EVC2*	c.3121-4G>T	splicing	Splicing mutation
	SNVs		c.157G>A	p.A53T	Missense mutation
	SNVs	*FGFR3*	c.490C>G	p.L164V	Missense mutation
	SNVs	*FMN1*	c.1945C>T	p.P649S	Missense mutation
	SNVs	*GAA*	c.2769C>A	p.V923V	Synonymous mutation
	SNVs	*GALC*	c.1832T>C	p.L611S	Missense mutation
	SNVs	*GIGYF2*	c.297T>C	p.A99A	Synonymous mutation
	SNVs		c.3612A>G	p.P1204P	Synonymous mutation
	SNVs	*GSN*	c.396C>T	p.T132T	Synonymous mutation
	SNVs	*HDAC4*	c.955G>A	p.A319T	Missense mutation
	SNVs	*HSPG2*	c.3794-3C>A	splicing	Splicing mutation
	SNVs	*LARS2*	c.1779G>C	p.L593L	Synonymous mutation
	SNVs	*MAPT*	c.418C>T	p.P140S	Missense mutation
	SNVs	*MYH11*	c.5696A>G	p.N1899S	Missense mutation
	SNVs	*OBSL1*	c.5108G>T	p.G1703V	Missense mutation
	SNVs	*PARK7*	c.535G>A	p.A179T	Missense mutation
	SNVs	*PRKAG2*	c.63C>G	p.G21G	Synonymous mutation
	SNVs	*PSEN2*	c.893T>C	p.M298T	Missense mutation
	SNVs	*RAI1*	c.840G>A[1]	p.Q280Q	Synonymous mutation
	SNVs	*RECQL4*	c.1115G>C	p.R372T	Missense mutation
	SNVs	*SCN1B*	c.351C>T	p.G117G	Synonymous mutation

	SNVs	*SCN3B*	c.390G>T	p.A130A	Synonymous mutation
	SNVs	*SCN5A*	c.3416G>A	p.R1139Q	Missense mutation
	SNVs		c.369G>A	p.A123A	Synonymous mutation
	SNVs	*SDHA*	c.1767C>T	p.V589V	Synonymous mutation
	SNVs	*SF1*	c.119C>T	p.A40V	Missense mutation
	SNVs	*UNC13A*	c.3100C>G	p.L1034V	Missense mutation
	INDELs	*AR*	c.171_173del	p.57_58del	non-frameshift mutation
	INDELs		c.1369_1377del	p.457_459del	non-frameshift mutation
	INDELs		c.1377_1378insGGC	p.G459delinsGG	non-frameshift mutation
	INDELs	*CTSA*	c.85_90del	p.29_30del	non-frameshift mutation
	INDELs	*GIGYF2*	c.3611_3612insGCA	p.P1204delinsPQ	non-frameshift mutation
	INDELs	*PRKAR1A*	c.273_274insAAAG	p.V91fs	Frameshift mutation
	INDELs	*RAI1*	c.832_837del	p.278_279del	non-frameshift mutation
	INDELs	*TBP*	c.163_171del	p.55_57del	non-frameshift mutation
Case 4	SNVs	*ADAMTSL2*	c.2142G>A	p.S714S	Synonymous mutation
	SNVs		c.2325C>G	p.S775S	Synonymous mutation
	SNVs	*AKAP9*	c.3827G>A	p.R1276Q	Missense mutation
	SNVs	*CIZ1*	c.1049C>T	p.A350V	Missense mutation
	SNVs	*CSHL1*	c.11C>T	p.T4M	Missense mutation
	SNVs	*CYP11B2*	c.111G>A	p.P37P	Synonymous mutation
	SNVs	*DYNC2H1*	c.8346G>A	p.M2782I	Missense mutation
	SNVs	*EIF2AK3*	c.2014G>A	p.E672K	Missense mutation
	SNVs	*FGFR3*	c.490C>G	p.L164V	Missense mutation
	SNVs	*GAA*	c.1758G>A	p.A586A	Synonymous mutation
	SNVs	*GALNS*	c.566+5T>C	splicing	Splicing mutation
	SNVs	*GIGYF2*	c.3445C>A	p.P1149T	Missense mutation
	SNVs	*GLB1*	c.363C>T	p.F121F	Synonymous mutation
	SNVs	*HTRA1*	c.176G>C	p.R59P	Synonymous mutation
	SNVs	*KCNH2*	c.902G>A	p.R301H	Missense mutation
	SNVs	*KCNJ16*	c.831C>T	p.N277N	Synonymous mutation
	SNVs	*MAN2B1*	c.748G>T	p.A250S	Missense mutation
	SNVs	*MYH7*	c.3918C>T	p.L1306L	Synonymous mutation
	SNVs	*NAGLU*	c.53C>T	p.A18V	Missense mutation
	SNVs	*PEX14*	c.208T>G	p.S70A	Missense mutation
	SNVs	*PTH1R*	c.18C>A	p.I6I	Synonymous mutation
	SNVs	*RAI1*	c.840G>A[1]	p.Q280Q	Synonymous mutation
	SNVs	*RECQL4*	c.1391-4G>T	splicing	Splicing mutation

	SNVs	*RYR2*	c.684C>T	p.L228L	Synonymous mutation
	SNVs	*SCN5A*	c.3416G>A	p.R1139Q	Missense mutation
	SNVs	*SETX*	c.7114G>A	p.D2372N	Missense mutation
	SNVs		c.867G>A	p.A289A	Synonymous mutation
	SNVs	*SH3PXD2B*	c.814G>A	p.A272T	Missense mutation
	SNVs	*SLC25A12*	c.125G>A	p.R42H	Missense mutation
	SNVs	*TNFRSF11A*	c.679G>T	p.A227S	Missense mutation
	SNVs	*VPS54*	c.404C>T	p.T135I	Missense mutation
	INDELs	*AR*	c.171_179del	p.57_60del	non-frameshift mutation
	INDELs		c.1369_1371del	p.457_457del	non-frameshift mutation
	INDELs	*RAI1*	c.832_837del	p.278_279del	non-frameshift mutation
	INDELs	*TBP*	c.163_171del	p.55_57del	non-frameshift mutation
Case 5	SNVs	*ACE*	c.460G>A	p.A154T	Missense mutation
	SNVs		c.1504G>T	p.V502L	Missense mutation
	SNVs	*ARSB*	c.658A>G	p.I220V	Missense mutation
	SNVs	*ATP13A2*	c.1980C>A	p.N660K	Missense mutation
	SNVs	*ATRX*	c.1492A>G[1]	p.R498G	Missense mutation
	SNVs	*BAG3*	c.898G>A	p.D300N	Missense mutation
	SNVs	*CEP290*	c.343A>G	p.N115D	Missense mutation
	SNVs	*CLN3*	c.852C>G	p.D284E	Missense mutation
	SNVs	*COL11A2*	c.4541G>A	p.R1514Q	Missense mutation
	SNVs	*COL5A1*	c.378G>T[1]	p.Q126H	Missense mutation
	SNVs	*CYP21A2*	c.1179C>G	p.H393Q	Missense mutation
	SNVs	*FMN1*	c.772A>G	p.K258E	Missense mutation
	SNVs	*NOTCH2*	c.137A>G	p.N46S	Missense mutation
	SNVs		c.112G>A	p.E38K	Missense mutation
	SNVs	*PRKRA*	c.5T>C	p.I2T	Missense mutation
	INDELs	*HOXD13*	c.168_179del	p.56_60del	non-frameshift mutation
	INDELs	*RAI1*	c.832_843	p.278_281del	non-frameshift mutation
Case 6	SNVs	*ABCC6*	c.3491G>A	p.R1164Q	Missense mutation
	SNVs		c.1773C>T	p.L591L	Synonymous mutation
	SNVs	*AKAP9*	c.5725G>A	p.A1909T	Missense mutation
	SNVs	*ALS2*	c.3517G>A	p.E1173K	Missense mutation
	SNVs	*ANK2*	c.3366C>T	p.N1122N	Synonymous mutation
	SNVs		c.3543C>T	p.A1181A	Synonymous mutation
	SNVs		c.4152T>C	p.D1384D	Synonymous mutation
	SNVs		c.6633C>T	p.A2211A	Synonymous mutation

	SNVs		c.7488A>G	p.T2496T	Synonymous mutation
	SNVs	*ARSA*	c.228C>T	p.A76A	Synonymous mutation
	SNVs	*ASAH1*	c.1059A>G	p.S353S	Synonymous mutation
	SNVs	*ATP6V0A2*	c.1590C>T	p.P530P	Synonymous mutation
	SNVs	*ATP7B*	c.3268G>A	p.V1090I	Missense mutation
	SNVs	*CLN3*	c.45G>A	p.E15E	Synonymous mutation
	SNVs	*DHCR24*	c.615C>T	p.S205S	Synonymous mutation
	SNVs	*DNMT1*	c.3622G>A	p.G1208S	Missense Mutation
	SNVs	*DSC2*	c.2019C>T	p.T673T	Synonymous mutation
	SNVs	*DSPP*	c.1326T>C	p.G442G	Synonymous mutation
	SNVs	*EVC*	c.1036C>T	p.L346L	Synonymous mutation
	SNVs	*FBN2*	c.1152G>A	p.T384T	Synonymous mutation
	SNVs	*FLNA*	c.2023-5G>A	splicing	Splicing mutation
	SNVs	*GALNT3*	c.1539T>G	p.G513G	Synonymous mutation
	SNVs	*GIGYF2*	c.3612A>G	p.P1204P	Synonymous mutation
	SNVs	*HDAC4*	c.955G>A	p.A319T	Missense mutation
	SNVs	*HSPG2*	c.11671+5G>A	splicing	Splicing mutation
	SNVs		c.7811G>A	p.S2604N	Missense mutation
	SNVs		c.4396-5C>T	splicing	Splicing mutation
	SNVs	*HTRA1*	c.151G>T	p.E51X	Nonsense mutation
	SNVs	*KCNJ16*	c.831C>T	p.N277N	Synonymous mutation
	SNVs	*LMBR1*	c.65C>T	p.T22M	Missense mutation
	SNVs	*MAN2B1*	c.719G>A	p.R240Q	Missense mutation
	SNVs	*MCOLN1*	c.876C>T	p.H292H	Synonymous mutation
	SNVs	*PCNT*	c.5710G>A	p.A1904T	Missense mutation
	SNVs		c.7413G>A	p.E2471E	Synonymous mutation
	SNVs	*PEX19*	c.129G>A	p.T43T	Synonymous mutation
	SNVs	*PRKAR1A*	c.496C>T	p.Q166X	Missense mutation
	SNVs	*RAI1*	c.840G>A[1]	p.Q280Q	Synonymous mutation
	SNVs	*SCN1B*	c.351C>T	p.G117G	Synonymous mutation
	SNVs	*SGCD*	c.51G>A	p.V17V	Synonymous mutation
	SNVs	*SLC11A2*	c.243G>T	p.L81L	Synonymous mutation
	SNVs	*SPAST*	c.311C>T	p.A104V	Missense mutation
	SNVs		c.1633-5T>A	splicing	Splicing mutaion
	SNVs	*TBXAS1*	c.211C>T	p.L71F	Missense mutation
	SNVs	*TREM2*	c.574G>A	p.A192T	Missense mutation
	SNVs	*WT1*	c.336C>T	p.G112G	Synonymous mutation

	INDELs	*AR*	c.170_171insGCAGCA	p.L57delinsLQQ	non-frameshift mutation
	INDELs		c.1369_1377del	p.457_459del	non-frameshift mutation
	INDELs	*FBXW4*	c.44_45insGGC	p.A15delinsAA	non-frameshift mutation
	INDELs	*GIGYF2*	c.3611_3612insGCA	p.P1204delinsPQ	non-frameshift mutation
	INDELs	*PRKAR1A*	c.569delG	p.W190fs	Frameshift mutation
	INDELs	*RAI1*	c.832_834del	p.278_278del	non-frameshift mutation
	INDELs	*TBP*	c.163_171del	p.55_57del	non-frameshift mutation
Case 7	SNVs	*ABCC6*	c.793A>G	p.R265G	Missense mutation
	SNVs	*ARHGAP31*	c.3983G>A	p.R1328Q	Missense mutation
	SNVs	*ARX*	c.565C>T	p.L189L	Synonymous mutation
	SNVs	*CACNA1S*	c.3606C>T	p.I1202I	Synonymous mutation
	SNVs	*CACNA1S*	c.2454G>T	p.A818A	Synonymous mutation
	SNVs	*CACNB2*	c.291A>G	p.E97E	Synonymous mutation
	SNVs	*CC2D2A*	c.501G>T	p.K167N	Missense mutation
	SNVs	*CLCN1*	c.2207C>T	p.T736I	Missense mutation
	SNVs	*COL2A1*	c.2065G>A	p.A689T	Missense mutation
	SNVs	*COL2A1*	c.372C>T	p.G124G	Synonymous mutation
	SNVs	*CYP21A2*	c.188A>T	p.H63L	Missense mutation
	SNVs	*DRD2*	c.561G>A	p.P187P	Synonymous mutation
	SNVs	*DSPP*	c.567C>T	p.S189S	Synonymous mutation
	SNVs	*EVC2*	c.1648G>A	p.A550T	Missense mutation
	SNVs	*FBN2*	c.3762C>T	p.D1254D	Synonymous mutation
	SNVs	*FGFR3*	c.1224C>G	p.S408S	Synonymous mutation
	SNVs	*FGFR3*	c.1481A>G	p.E494G	Missense mutation
	SNVs	*FIG4*	c.1728A>G	p.R576R	Synonymous mutation
	SNVs	*GAA*[1]	c.447G>A	p.T149T	Synonymous mutation
	SNVs	*HEXA*	c.200G>A	p.R67H	Missense mutation
	SNVs	*HPGD*	c.476A>G	p.N159S	Missense mutation
	SNVs	*HTRA1*	c.1221C>T	p.D407D	Synonymous mutation
	SNVs	*JPH2*	c.780C>T	p.A260A	Synonymous mutation
	SNVs	*KCNJ12*	c.1113C>G	p.S371R	Missense mutation
	SNVs	*KCNJ16*[1]	c.831C>T	p.N277N	Synonymous mutation
	SNVs	*LRP5*	c.2907C>T	p.S969S	Synonymous mutation
	SNVs	*LRRK2*	c.4883G>C	p.R1628P	Synonymous mutation
	SNVs	*MYH7*	c.5704G>C	p.E1902Q	Missense mutation
	SNVs	*PEX10*	c.27G>A	p.P9P	Synonymous mutation
	SNVs	*PEX16*	c.803G>A	p.R268Q	Missense mutation

	SNVs	*PIGV*	c.572C>G	p.T191S	Missense mutation
	SNVs	*PITX1*	c.42G>T	p.P14P	Synonymous mutation
	SNVs	*PRKAR1A*	c.366C>G	p.Y122X	Nonsense mutation
	SNVs	*PRKAR1A*	c.678C>T	p.I226I	Synonymous mutation
	SNVs	*PSEN1*	c.654A>G	p.P218P	Synonymous mutation
	SNVs	*RAI1*	c.840G>A	p.Q280Q	Synonymous mutation
	SNVs	*SGCD*	c.845A>G	p.Q282R	Missense mutation
	SNVs	*SMPD1*	c.1598C>T	p.P533L	Missense mutation
	SNVs	*SOST*	c.56G>A	p.R19H	Missense mutation
	SNVs	*TP63*	c.577C>T	p.L193L	Synonymous mutation
	INDELs	*RAI1*	c.832_834del	p.278_278del	non-frameshift mutation
	INDELs		c.834_835insCAA	p.Q278delinsQQ	non-frameshift mutation
	INDELs	*TAF15*	c.1495_1515del	p.499_505del	non-frameshift mutation
	INDELs	*TBP*	c.163_171del	p.55_57del	non-frameshift mutation

Note: NGS = targeted next generation sequencing, CM = cardiac myxoma, SNVs = Single nucleotide variations, INDELs = Insertion-deletions.
